# Genomic inbreeding measures applied to a population of mice divergently selected for birth weight environmental variance

**DOI:** 10.3389/fgene.2023.1303748

**Published:** 2023-12-14

**Authors:** Candela Ojeda-Marín, Isabel Cervantes, Nora Formoso-Rafferty, Juan Pablo Gutiérrez

**Affiliations:** ^1^ Departamento de Producción Animal, Facultad de Veterinaria, Universidad Complutense de Madrid, Madrid, Spain; ^2^ Departamento de Producción Agraria, E.T.S. Ingeniería Agronómica, Alimentaria y de Biosistemas, Universidad Politécnica de Madrid, Madrid, Spain

**Keywords:** genomic inbreeding, effective population size, divergent selection, birth weight environmental variability, increase in inbreeding

## Abstract

This study aimed to compare different inbreeding measures estimated from pedigree and molecular data from two divergent mouse lines selected for environmental birth weight during 26 generations. Furthermore, the performance of different approaches and both molecular and pedigree data sources for estimating *Ne* were tested in this population. A total of 1,699 individuals were genotyped using a high-density genotyping array. Genomic relationship matrices were used to calculate molecular inbreeding: Nejati-Javaremi (*F*_*NEJ*_), Li and Horvitz (*F*_*L&H*_), Van Raden method 1 (*F*_*VR1*_) and method 2 (*F*_*VR2*_), and Yang (*F*_*YAN*_). Inbreeding based on runs of homozygosity (*F*_*ROH*_) and pedigree inbreeding (*F*_*PED*_) were also computed. *F*_*ROH*_, *F*_*NEJ*_, and *F*_*L&H*_ were also adjusted for their average values in the first generation of selection and named *F*_*ROH0*_, *F*_*NEJ0*_, and *F*_*L&H0*_. *∆F* was calculated from pedigrees as the individual inbreeding rate between the individual and his parents (*∆F*_*PEDt*_) and individual increases in inbreeding (*∆F*_*PEDi*_). Moreover, individual *∆F* was calculated from the different molecular inbreeding coefficients (*∆F*_*NEJ0*_, *∆F*_*L&H*_, *∆F*_*L&H0*_, *∆F*_*VR1*_, *∆F*_*VR2*_, *∆F*_*YAN*_, and *∆F*_*ROH0*_)*.* The *Ne* was obtained from different *∆F*, such as *Ne*_*PEDt*_, *Ne*_*PEDi*_, *Ne*_*NEJ0*_, *Ne*_*L&H*_, *Ne*_*L&H0*_, *Ne*_*VR1*_, *Ne*_*VR2*_, *Ne*_*YAN*_, and *Ne*_*ROH0*_. Comparing with *F*_*PED*_ *, F*_*ROH*_ *, F*_*NEJ*_ and *F*_*VR2*_ overestimated inbreeding while *F*_*NEJ0*_ *, F*_*L&H*_ *, F*_*L&H0*_ *, F*_*VR1*_ and *F*_*YAN*_ underestimated inbreeding. Correlations between inbreeding coefficients and *∆F* were calculated. *F*_*ROH*_ had the highest correlation with *F*_*PED*_ (0.89); *F*_*YAN*_ had correlations >0.95 with all the other molecular inbreeding coefficients. *Ne*_*PEDi*_ was more reliable than *Ne*_*PEDt*_ and presented similar behaviour to *Ne*_*L&H0*_ and *Ne*_*NEJ0*_. Stable trends in *Ne* were not observed until the 10th generation. In the 10th generation *Ne*_*PEDi*_ was 42.20, *Ne*_*L&H0*_ was 45.04 and *Ne*_*NEJ0*_ was 45.05 and in the last generation these Ne were 35.65, 35.94 and 35.93, respectively *F*_*ROH*_ presented the highest correlation with *F*_*PED*_, which addresses the identity by descent probability (IBD). The evolution of *Ne*_*L&H0*_ and *Ne*_*NEJ0*_ was the most similar to that of *Ne*_*PEDi*_. Data from several generations was necessary to reach a stable trend for *Ne*, both with pedigree and molecular data. This population was useful to test different approaches to computing inbreeding coefficients and *Ne* using molecular and pedigree data.

## 1 Introduction

Inbreeding appears due to mating between related individuals and is related to negative consequences because of an increase in homozygosity, such as a reduction in fitness, namely, inbreeding depression (Hedrick, 2012; Alemu et al., 2021). Therefore, the study of inbreeding is essential in many areas, e.g., animal and plant breeding (Villanueva et al., 2021), human genetics (McQuillan et al., 2012), and evolutionary (Roff, 1997) and conservation biology (Frankham et al*.,* 2010).

Traditionally, pedigree data has been used to measure inbreeding (Howard et al., 2017; Villanueva et al., 2021); however, this only provides the expected proportions of the genome that are identical by descent (IBD) and does not capture variation due to Mendelian sampling and linkage during gamete formation (Howard et al., 2017).

The implementation of molecular technologies has led to the development of numerous genomic estimators of inbreeding coefficients (Howard et al., 2017; Alemu et al., 2021; Villanueva et al., 2021), which can distinguish between individuals with the same ascendants and differentiate inbreeding at specific regions in chromosomes (Howard et al., 2017; Villanueva et al., 2021). Different approaches have been developed to measure inbreeding coefficients based on genomic data, including methods based on homozygous stretches of DNA sequences (runs of homozygosity—ROH) (Ceballos et al., 2018). However, ROH detection is highly dependent on the parameters set by the user (Peripolli et al., 2017; Rodríguez-Ramilo et al*.,* 2019; Villanueva et al., 2021); therefore, comparisons between studies are not straightforward. In this context, other methodologies appeared that detected homozygosity by descent (HBD) segments under a probabilistic framework based on hidden Markov models. The probability of a single nucleotide polymorphism (SNP) being in an HBD segment was modelled as a function of genotyping error, intermarker distances and allele frequencies (Druet and Gautier, 2017). In addition, other methods are based on analysing each SNP and are derived from genomic relationship matrices (GRMs) (Li and Horvitz, 1953; Nejati-Javaremi et al., 1997; VanRaden, 2008; Yang et al., 2010; VanRaden et al., 2011). GRMs are used to obtain genomic predictions in animal breeding and can be used to estimate inbreeding given (Villanueva et al., 2021). These measures, however, are designed with different constraints and can lead to very different results with several implications (Villanueva et al., 2021).

One of the indicators most used to assess the genetic diversity of a population is the effective population size (*Ne*) (Wright, 1931). There are some approaches based on different indicators that have been used to measure *Ne*: the change in allele frequencies across generations (variance *Ne*), increase in homozygosity (inbreeding *Ne*), increase in kinship (coancestry *Ne*) or the rate of loss of less frequent alleles (eigenvalue *Ne*). *Ne* can also be estimated using census parameters, pedigree data, individual genotypes or demographic information (Sjödin et al., 2005; Leroy et al., 2013). The relationship between the increase in inbreeding (*∆F*) and *Ne* is defined by the classic formula: *Ne* $=\frac{1}{2∆F}$ . Traditionally, *∆F* has been calculated as the increase in the inbreeding rate between two successive generations (Wright, 1931). However, other methods have been proposed to estimate the individual increase in inbreeding based on pedigree knowledge, which has demonstrated less susceptibility to mating method changes (Gutiérrez et al., 2008; Gutiérrez et al*.,* 2009; Cervantes et al., 2011). Moreover, the availability of molecular information has led to the estimation of molecular *Ne*. Some estimators have been proposed based in heterozygosity excess (Pudovkin et al., 1996), linkage disequilibrium (Hill, 1981), temporal changes in allele frequency (Krimbas and Tsakas, 1971; Nei and Tajima, 1981; Pollak, 1983), half-sib and full-sib (Wang, 2009), or in approximate Bayesian computation that take into account, among other parameters, the number of alleles per locus, the linkage disequilibrium or the mean and variance of multilocus homozygosity (Tallmon et al*.,* 2008). Wang et al*.,* 2016 discussed some concerns about these different *Ne* estimators as the reliability, the interpretation of each method, or the implicit assumption in some of these methods.

A divergent selection experiment for birth weight variability in mice has been successfully carried out, creating two lines, one homogeneous and the other heterogeneous over 26 generation (Formoso-Rafferty et al., 2016a). The experimental mating design avoided sharing grandparents and was optimised as a function of selection criteria in both selected lines (Formoso-Rafferty et al., 2016b). The homogeneous line presented advantages in production, animal welfare, response to selection, and traits related to robustness, such as longevity, survival and feed efficiency (Formoso-Rafferty et al., 2017; Formoso-Rafferty et al., 2018; Formoso-Rafferty et al., 2019; Formoso-Rafferty et al., 2020; Formoso-Rafferty et al., 2022; Formoso-Rafferty et al., 2023).

This study aimed to compare different inbreeding measures estimated from pedigree and molecular data from divergent mouse lines for environmental variability in birth weight. Furthermore, the performance of *Ne* estimated using different approaches and both molecular and pedigree data sources was tested in this divergent population. The applications of different methodologies in this particular population structure (strong divergent selection) are intended to evaluate the suitability in populations under selection.

## 2 Methods

Pedigree data were obtained from 26 discrete generations of a successful divergent selection experiment for environmental variability of birth weight in mice, including an additional five previous discrete generations randomly mated. In the experiment, in each generation, 43 females were mated to one male to give a maximum of two parturitions, avoiding mating animals sharing grandparents, with an approximately 30% proportion selected within the line. In the five preceding generations, 32 males were mated to 64 females, each male with 2 females. More details of the selection process can be found in Formoso-Rafferty et al. (2016a).

A total of 1824 individuals from the 26 selection generations were genotyped using the Affymetrix Mouse Diversity Genotyping Array with 616,137 SNPs. The individuals genotyped were those selected according to the selection criteria in each generation. During quality control (QC) animals with a call rate lower than 97% were removed, leaving 1,669 for analysis. Of these, 1,587 were females and 112 were males. The first generation of selected mice was used as the reference population (a total of 70 individuals, all females). The QC was also applied to the SNPs: 3% of missing genotypes were allowed, SNPs mapped to sex chromosomes were removed, and 545,656 SNPs were retained. This set was used to detect ROH to ensure maximum genome coverage and, therefore, avoid loss of information: no minor allele frequency (MAF) was applied as recommended other authors recommended (Meyermans et al., 2020).

Additional QC was applied to determine the other genomic inbreeding coefficients estimators: SNPs presenting MAFs lower than 0.05 in the reference population were removed. Additionally, remnant SNPs with MAFs less than 0.05 among the whole population were also removed. Finally, 173,546 SNPs were kept, which were used to obtain inbreeding coefficients from different GRMs. The number of animals genotyped per generation in the selected population is presented in Table 1.

**TABLE 1:** Number of animals sampled per line and generation. H-Line: high variability line. L-Line: low variability line.

Generation	H-line	L-line
1	36	34
2	41	36
3	35	39
4	37	36
5	29	36
6	33	32
7	33	33
8	37	39
9	27	35
10	30	34
11	34	26
12	31	30
13	33	32
14	28	30
15	29	32
16	32	30
17	33	32
18	15	22
19	25	29
20	28	27
21	71	67
22	30	26
23	22	27
24	24	27
25	34	25
26	35	41

### 2.1 Inbreeding coefficients

Inbreeding coefficients from genomic data were obtained from the diagonal elements of different GRMs and ROH.

Inbreeding based on GRMs was named using the nomenclature chosen by Villanueva et al*.,* (2021); therefore, inbreeding coefficients were calculated as follows:

1) *F*_*NEJ*_ uses the diagonal elements of the allelic relationship matrix of Nejati-Javaremi et al. (1997). *F*_*NEJ*_ is calculated using all individuals (*F*_*NEJT*_), those from the high variability line (*F*_*NEJH*_) and those from the low variability line (*F*_*NEJL*_).
2) $F_{{NEJ}_{0}}$ of an individual is calculated as $F_{{NEJ}_{0}}=\frac{F_{NEJ-}\bar{F_{{NEJ}_{1st}}}}{1-F_{{NEJ}_{1st}}}$ , where $F_{NEJ}$ is the Nejati-Javaremi inbreeding coefficient and $\bar{F_{{NEJ}_{1st}}}$ is the average Nejati-Javaremi inbreeding coefficient of the reference population.
3) *F*_*L&H*_ estimates the deviation of the observed frequency of homozygotes from the expected frequency of homozygotes in the reference population under Hardy-Weinberg proportions (Li and Horvitz, 1953).
4) $F_{{L\&H}_{0}}$ of an individual *k* is calculated as $F_{{L\&H}_{0}}=\frac{F_{L\&H-}\bar{F_{{L\&H}_{1st}}}}{1-F_{{L\&H}_{1st}}}$ , where $F_{L\&H}$ is the Li and Horvitz inbreeding coefficient of the individual and $\bar{F_{{L\&H}_{1st}}}$ is the average *F*_*L&H*_ of the reference population*.* Therefore, $F_{{L\&H}_{0}}$ is expected to perform similarly to that of $F_{{NEJ}_{0}}$ .
5) *F*_*VR1*_ is computed from the diagonal elements of the GRM obtained according to Van Raden’s method 1 (VanRaden, 2008).
6) *F*_*VR2*_ is computed from the diagonal elements of the GRM obtained according to Van Raden’s method 2 (Leutenegger et al*.,* 2003; VanRaden, 2008).
7) *F*_*YAN*_ uses the diagonal elements from the GRM of Yang to compute inbreeding coefficients (Yang et al., 2010).
8) Sliding window algorithms were used to detect ROH in each population. The parameters set were as follows: 50 SNPs per window; one heterozygote allowed in a window; no limit of the number of heterozygotes per ROH; five missing SNPs allowed in a window; the minimum length of an ROH was 1,000 kb; the minimum number of homozygous SNPs in an ROH was set at 100; the required minimum density was set at one SNP/50 kb; the window threshold was set at 0.5; and the minimum distance between two ROHs was 1,000 kb. Inbreeding based on ROH was calculated as $F_{{ROH}_{i}}$ (McQuillan et al.*,* 2012).
9) $F_{{ROH}_{0}}$ is calculated as $F_{{ROH}_{0}}=\frac{F_{ROH-}\bar{F_{{ROH}_{1st}}}}{1-\bar{F_{{ROH}_{1st}}}}$ , where $F_{\text{ROH}}$ is the ROH inbreeding coefficient and $\bar{F_{{ROH}_{1st}}}$ is the average ROH inbreeding coefficient of the reference population.
10) Pedigree inbreeding coefficients (*F*_*PED*_) are defined as the probability of an individual having two identical alleles by descent and are computed following Meuwissen and Luo (1992).

In summary, we tested several methods; some were intrinsically adjusted for the allele frequencies of the reference population (*F*_*L&H*_, *F*_*VR1*_, *F*_*VR2*_, and *F*_*YAN*_), others were adjusted for mean inbreeding of the reference population ( $F_{{NEJ}_{0}}$ , $F_{{L\&H}_{0}}$ , and $F_{{ROH}_{0}}$ ), and, finally, some were not adjusted (*F*_*PED*_, *F*_*NEJ*_, and *F*_*ROH*_). The genomic inbreeding estimators described above were chosen because they have been already tested in other populations (Alemu et al.*,* 2020; Villanueva et al*.,* 2021; Caballero et al., 2022), except for the adjusted inbreeding estimators 2, 4 and 9, which, not been previously described before. The application of these estimators to this population with a high number of generations, a particular and very structure linked to a specific mating design, represented an opportunity to evaluate the performance of these inbreeding coefficient estimators.

Expected homozygosity (*F*_*EXP*_) was calculated from the frequency of the reference allele (*p*) per generation as $F_{\mathrm{EXP}}=1-2p\left(1-p\right)$ . This coefficient was calculated considering both lines together (*F*_*EXP*_) and within the high variability line (*F*_*EXPH*_) and the low variability line (*F*_*EXPL*_).

Our own R code was used to calculate coefficients based on GRMs. PLINK v 1.9 (Chang et al., 2015) was used to detect ROH, and ENDOG v 4.8 (Gutierrez and Goyache, 2005) was used to calculate pedigree inbreeding coefficients.

### 2.2 Individual increase in inbreeding and realised effective population size

Effective population size was computed for each generation from individual increases in inbreeding (Gutiérrez et al., 2008; Gutiérrez et al., 2009) based on the different inbreeding coefficients described above. When pedigree inbreeding was used, two approaches were applied to calculate the individual increase in inbreeding. One is based on the classic formula: ${\Delta F}_{PEDt}=\frac{F_{{PED}_{t}}-F_{{PED}_{t-1}}}{1-F_{{PED}_{t-1}}}$ , where $F_{{PED}_{t}}$ is the inbreeding coefficient of the individual of generation *t* and $F_{{PED}_{t-1}}$ is the average coefficient of inbreeding of their parents (Falconer and Mackay, 1996). Then, the effective population size was computed as ${Ne}_{PEDt}=\frac{1}{2\bar{∆F_{PED}}}$ , where $\bar{∆F_{PEDt}}$ is the average *ΔF*_*PEDt*_ of *n* individuals included in each generation (Falconer and Mackay, 1996).

Moreover, the individual increase in inbreeding coefficient using *F*_*PED*_ was also calculated as ${\Delta F}_{PEDi}=1-\sqrt[{t-1}]{1-F_{PEDi}}$ (Gutiérrez et al., 2009), where *F*_*PEDi*_ is the individual coefficient of inbreeding of *i* and *t* are the number of generations. The individual increase in inbreeding has been proposed as a measure of standardized inbreeding rate per generation (Gonzále-Recio et al., 2007). The realised effective population was computed as ${Ne}_{PEDi}=\frac{1}{2\bar{∆F_{PEDi}}}$ , where $\bar{∆F_{PEDi}}$ is the average *ΔF*_*PEDi*_ of *n* individuals included in each generation (Cervantes et al., 2008).

The molecular *N*_*e*_ based on *F*_*NEJ0*_ ( ${Ne}_{NEJ0}),$ *F*_*L&H0*_ $\left({Ne}_{L\&H0}\right.$ ), *F*_*VR1*_ ( $\left.{Ne}_{VR1}\right)$ , *F*_*VR2*_ ( ${Ne}_{VR2}$ ), *F*_*YAN*_ ( ${Ne}_{YAN}$ ), and *F*_*ROH0*_ ( $\left.{Ne}_{ROH0}\right)$ was calculated as $Ne=\frac{1}{2∆F}$ , where ∆F is the average *ΔFi* calculated from *F*_*NEJ0*_, *F*_*L&H0*_, *F*_*VR1*_, *F*_*VR2*_, *F*_*YAN*_, or *F*_*ROH0*_ of *n* individuals included in each generation. *ΔFi* was calculated as: $∆Fi=1-\sqrt[{t}]{1-F_{i}}$ , where *F*_*i*_ is the individual *F*_*NEJ0*_, *F*_*L&H0*_, *F*_*ROH0*_, *F*_*VR1*_, *F*_*VR2*_ or *F*_*YAN*_ coefficient of inbreeding of individual *i* and *t* is the number of generations that passed since the first generation of selection where the reference population has been defined.

The expected *N*_*e*_ per generation was computed as ${Ne}_{\mathrm{EXP}}=\frac{1}{2∆F_{\mathrm{EXP}}}$ , where ${∆F}_{\mathrm{EXP}}$ is the expected increase in homozygosity per generation calculated as $∆F_{\mathrm{EXP}}=\frac{F_{{\mathrm{EXP}}_{t}}-F_{{\mathrm{EXP}}_{t-1}}}{1-F_{{\mathrm{EXP}}_{t-1}}}$ , where $F_{{\mathrm{EXP}}_{t}}$ is the expected homozygosity of the current generation and $F_{{\mathrm{EXP}}_{t-1}}$ is the average coefficient of expected homozygosity of the previous generation. This expected *N*_*e*_ was obtained considering both lines together $\left({Ne}_{\mathrm{EXP}}\right)$ , also within line for the high variability line $\left({Ne}_{EXPH}\right)$ and for the low variability line $\left({Ne}_{EXPH}\right)$ .

In addition, the number of generations that passed since the founder generation to the reference population could be derived from the classic formula $F_{t}={1-\left(1-∆F\right)}^{t}$ , where *F*_*t*_ is the average inbreeding of the reference population and *t* is the generation of the reference population. Thus, the estimated generations that had passed from the founder population to the reference population are $t=\frac{\ln\left(1-Ft\right)}{\ln\left(1-∆F\right)}$ . Different *t* values were calculated considering *F*_*NEJ*_ (*t*_*NEJ*_) and *F*_*ROH*_ (*t*_*ROH*_); *t*_*NEJ*_ and *t*_*ROH*_ were calculated using the average inbreeding coefficients of the reference population, and the increase in inbreeding ( $∆F=\frac{1}{2Ne}$ ) was derived from *Ne* based on the sex ratio (*Ne*_*s*_), which was calculated as $Nes=\frac{4MF}{M+F}$ , where *M* is the number of breeding males and *F* is the number of breeding females (Wright, 1931). *Ne*_*s*_ was calculated using the permanent number of males (32) and females (64) in the randomly mated population, which was the origin of the selected divergent lines.

Moreover, *t* was also calculated using the average *F*_*PED*_ of the reference population (*t*_*PED*_) and the average *∆F*_*PEDi*_ of whole selection generation*s*. In this case*, t*_*PED*_ represented the number of generations elapsed if the breeding method of the selection experiment would be applied from the founder population of the recorded pedigree to the first generation of the experiment (reference population).

Pearson’s correlation coefficients were calculated between all computed inbreeding coefficients and increases in inbreeding. Correlations between the different inbreeding coefficients were calculated for the whole population and different generation groups: initial (1, 2, 3, and 4), intermediate (16, 17, 18, and 19), and most recent generations (23, 24, 25, and 26). R software was used to calculate correlations using the function “cor·” (Rizopoulos, 2007).

## 3 Results

**FIGURE 1:**
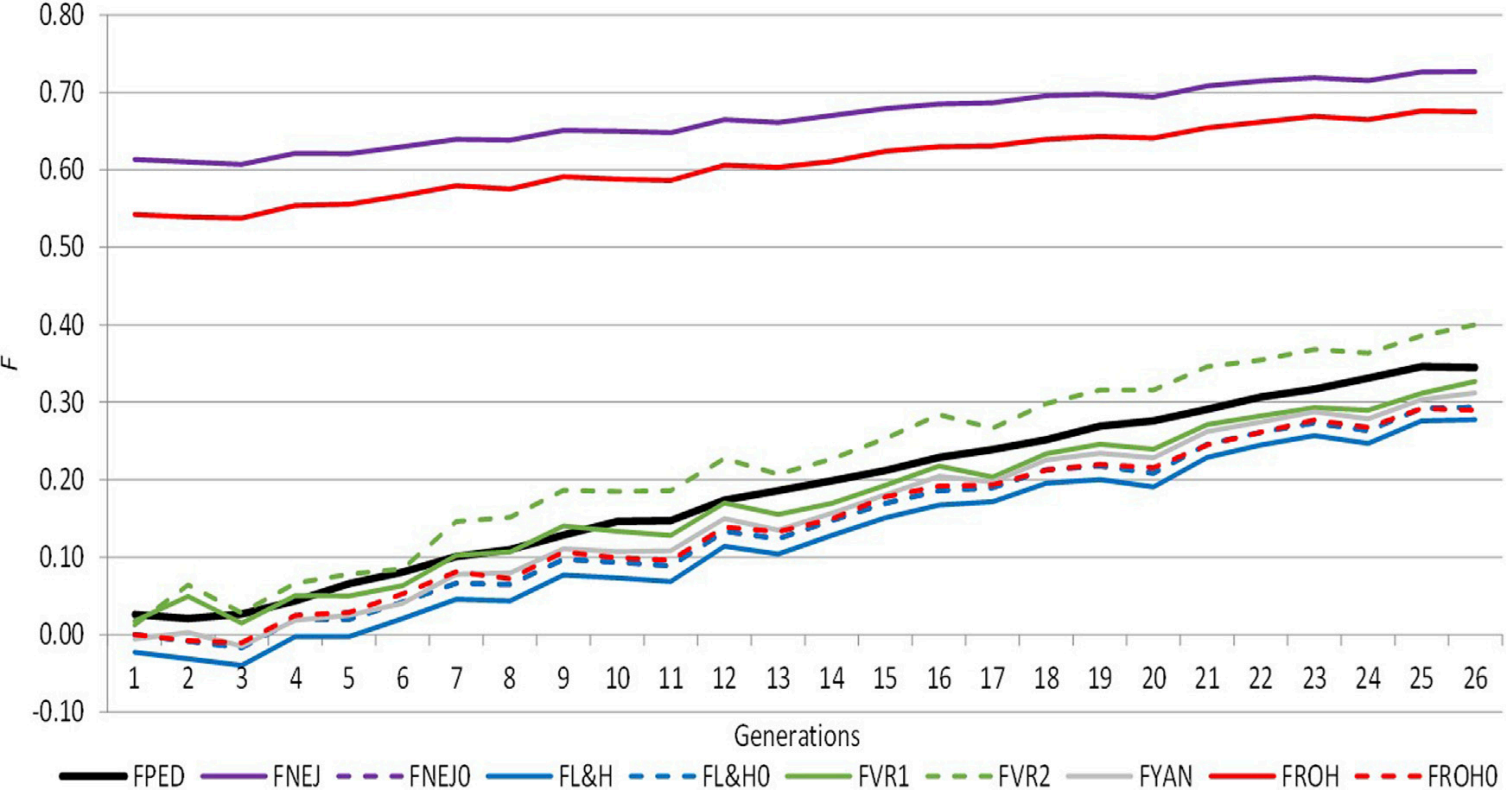
Evolution of the different inbreeding coefficients computed from pedigree and molecular information across the selection generation. *F*_*PED*_: inbreeding coefficient computed from pedigree information. *F*_*NEJ*_: inbreeding coefficient computed from the diagonal of the Nejati Javaremi Matrix. *F*_*L&H*_: inbreeding coefficient computed from the diagonal of the Li and Horvitz Matrix. *F*_*VR1*_: inbreeding coefficient computed from the diagonal of the Van Raden type 1 matrix. *F*_*VR2*_: inbreeding coefficient computed from the diagonal of the Van Raden type 2 matrix. *F*_*YAN*_: inbreeding coefficient computed from the diagonal of the Yang matrix. *F*_*ROH*_: inbreeding coefficient computed using runs of homozygosity. *F*_*PED0*_, *F*_*NEJ0*_, *F*_*L&H0*_, and *F*_*ROH0*_ were adjusted for the mean inbreeding coefficients of the first selection generation.

Figure 1 shows the trend of average inbreeding across generations calculated using molecular and pedigree approaches, which was positive in both cases. Both approaches presented similar performances across generations, except *F*_*ROH*_ and *F*_*NEJ*_, which showed the greatest amount of inbreeding in the first and last generations. In the first generation, *F*_*ROH*_ was 0.54 and *F*_*NEJ*_ was 0.61, and in the last generation, *F*_*ROH*_ was 0.68 and *F*_*NEJ*_ was 0.73. The lowest individual inbreeding coefficient was identified for *F*_*L&H*_ in the first (−0.02) and last generation (0.28) (Supplementary Table S1). There were not differences in the evolution of the different inbreeding coefficients in the two selection lines when analysed separately (results are not shown). We therefore decided to analyse the data together.

**FIGURE 2:**
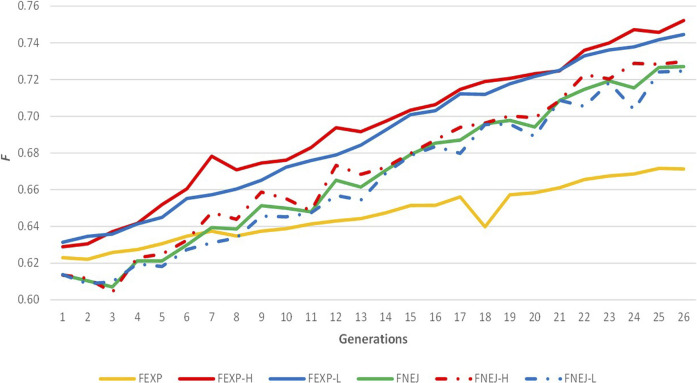
Evolution of expected homozygosity (*F*_*EXP*_) and mean inbreeding coefficient obtained from the Nejati-Javaremi genomic relationship matrix (*F*_*NEJ*_) across the selection generations. *F*_*EXP-T*_ was calculated using all individuals. *F*_*EXP-H*_ was calculated using individuals from the high variability line. *F*_*EXP-L*_ was calculated using individuals from the low variability line. *F*_*NEJ-T*_ was calculated using all genotyped individuals. *F*_*NEJ-H*_ was calculated using individuals from the high variability line. *F*_*NEJ-L*_ was calculated using individuals from the low variability line.

The evolution of expected homozygosity and *F*_*NEJ*_ for the whole population and selected lines for divergent birth weight variability across generations are represented in Figure 2. Expected homozygosity presented a positive trend when data from all individuals was used and when selected lines were analysed separately. Moreover, *F*_*EXPH*_ and *F*_*EXPL*_ were similar to *F*_*NEJH*_ and *F*_*NEJL*_, with 0.75 *F*_*EXPH*_, 0.74 *F*_*EXPL*_, 0.73 *F*_*NEJH*_, and 0.72 *F*_*NEJL*_ in the last generation. *F*_*EXP*_ was greater than *F*_*NEJ*_ until the seventh generation. However, after these generations, *F*_*EXP*_ was markedly lower than *F*_*NEJ*_ and lower than expected homozygosity within line because of the structure created by divergent selection. The highest values of *F*_*EXP*_ and *F*_*NEJ*_ were reached in the last selection generation, at 0.67 and 0.72, respectively.

Correlations between molecular and pedigree inbreeding coefficients using the whole population are shown in Table 2. All correlations were greater than 0.84. The correlations between *F*_*PED*_ and molecular inbreeding coefficients were between 0.84 (*F*_*PED*_-*F*_*VR1*_) and 0.89 (*F*_*PED*_-*F*_*ROH*_). The strongest correlations between molecular inbreeding coefficients were detected between *F*_*NEJ*_ and *F*_*L&H*_ (1.00). The *F*_*YAN*_ showed correlations greater than 0.95 with all other molecular inbreeding coefficients, and the correlation with *F*_*PED*_ was 0.88. When only a few generations were used to calculate the correlation coefficients, these correlations were lower between *F*_*PED*_ and the molecular inbreeding coefficients (Supplementary Table S2). In the initial generations, *F*_*PED*_ showed the highest correlation with *F*_*ROH*_ (0.25) and the lowest correlation with *F*_*VR1*_ (0.11)*.* However*,* in the intermediate generations, *F*_*PED*_ showed the highest correlations with *F*_*NEJ*_ and *F*_*L&H*_ (0.39), and the lowest correlation was observed with *F*_*VR2*_ (0.22). Moreover, in the most recent generations, the highest correlations were also observed between *F*_*PED*_-*F*_*NEJ*_ and *F*_*PED*_-*F*_*L&H*_ (0.22), and the lowest correlation was observed between *F*_*PED*_-*F*_*VR2*_ (0.13). Finally, in general, *F*_*YAN*_ showed the highest correlations with other molecular inbreeding coefficients in the initial, intermediate, and final generations.

**TABLE 2:** Correlation coefficients between molecular and pedigree inbreeding coefficients of all genotyped individuals. *F*_*PED*_: inbreeding coefficient computed from pedigree. *F*_*NEJ*_: inbreeding coefficient computed from the diagonal of the Nejati-Javaremi matrix. *F*_*L&H*_ i: inbreeding coefficient computed from the diagonal of Li and Horvitz Matrix. *F*_*VR1*_: inbreeding coefficient computed from the diagonal of the Van Raden type 1 matrix. *F*_*VR2*_: inbreeding coefficient computed from the diagonal of the Van Raden type 2 matrix. *F*_*YAN*_: inbreeding coefficient computed from the diagonal of the Yang matrix. *F*_*ROH*_: inbreeding coefficient computed using runs of homozygosity.

	*F*_*PED*_	*F*_*NEJ*_	*F*_*L&H*_	*F*_*VR1*_	*F*_*VR2*_	*F*_*YAN*_	*F*_*ROH*_
*F*_*PED*_	1.00	0.88	0.88	0.84	0.85	0.88	0.89
*F*_*NEJ*_		1.00	1.00	0.91	0.87	0.96	0.98
*F*_*L&H*_			1.00	0.91	0.87	0.96	0.98
*F*_*VR1*_				1.00	0.98	0.98	0.89
*F*_*VR2*_					1.00	0.96	0.85
*F*_*YAN*_						1.00	0.94
*F*_*ROH*_							1.00

**FIGURE 3:**
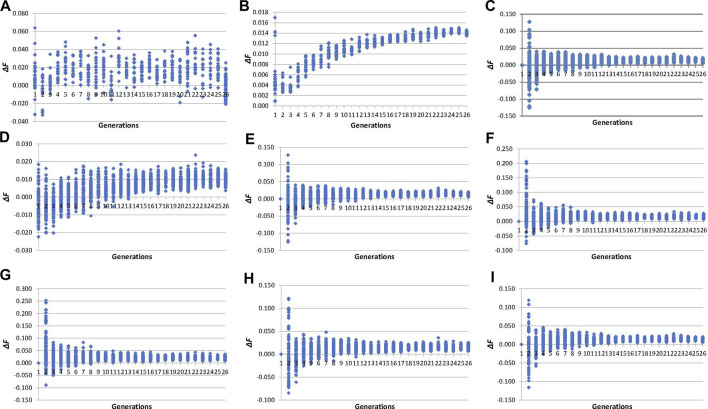
Evolution across selection generations of molecular and pedigree increases in inbreeding coefficients (*∆F*) of all genotyped individuals. **(A)** *: ∆F*_*PEDt*_ derived from pedigree referenced against the inbreeding coefficient of the individual’s parents. **(B)** *: ∆F*_*PEDi*_ derived from pedigree referred to the number of generations of the individual. **(C)**: *∆F*_*NEJ0*_ derived from inbreeding coefficients (*F*) of the Nejati-Javaremi matrix (*F*_*NEJ*_) adjusted for the mean *F*_*NEJ*_ of the first selection generation. **(D)**: *∆F*_*L&H*_ derived from the diagonal of the Li and Horvitz matrix. **(E)**: *∆F*_*L&H0*_ derived from *F* of the Li and Horvitz matrix (*F*_*L&H*_) adjusted for the mean *F*_*L&H*_ of the first selection generation. **(F)**: *∆F*_*VR1*_ derived from the diagonal of the Van Raden type 1 matrix. **(G)**: *∆F*_*VR2*_ derived from the diagonal of the Van Raden type 2 matrix. **(H)**: *∆F*_*YAN*_ derived from the diagonal of the Yang matrix. **(I)**: *∆F*_*FROH0*_ derived from *F* of runs of homozygosity (*F*_*ROH*_) adjusted for the mean *F*_*ROH*_ of the first selection generation.

The evolution of individual inbreeding increases across generations calculated by pedigree and molecular inbreeding coefficients are shown in Figure 3. The *∆F*_*PEDt*_ (values between −0.03 and 0.06, Figure 3A) showed an irregular evolution across generations unlike ∆*F*_*PEDi*_ (0.00 and 0.02, Figure 3B), which increased during fifteen generations and became stable thereafter. Individual increases in inbreeding calculated from molecular inbreeding coefficients showed a similar trend, with higher variability among individuals until the 10th generation, after which the trend was stabilised. The ∆*F*_*L&H0*_ (values between −0.13 and 0.13, Figure 3E) performed more like the other molecular increases in inbreeding (*∆F*_*NEJ0,*_ *∆F*_*VR1,*_ *∆F*_*VR2,*_ *∆F*_*YAN*_ and *∆F*_*ROH0*_) than *∆F*_*L&H*_ (values between −0.02 and 0.02, Figure 3D) in the first five generations of selection.

Table 3 shows the correlations between increases in inbreeding calculated using all generations. Correlations greater than 0.90 were identified between *∆F*_*L&H0*_ *—∆F*_*NEJ0*_ (1.00), *∆F*_*L&H*_ *—∆F*_*YAN*_ (0.92), *∆F*_*ROH0*_ *—∆F*_*NEJ0*_ (0.91), and *∆F*_*ROH0*_ *—∆F*_*L&H0*_ (0.91). Moreover, *∆F*_*YAN*_ was strongly correlated with *∆F*_*VR1*_ (0.89) and *∆F*_*VR2*_ (0.86). The lowest correlations were detected between *∆F*_*PEDt*_ and all other inbreeding increases being between 0.38 for *∆F*_*PEDi*_ and 0.18 for *∆F*_*VR1*_ and *∆F*_*VR2*_. Interestingly, higher correlations were detected between *∆F*_*L&H*_ and *∆F*_*YAN*_, *∆F*_*VR1*_ and *∆F*_*VR2*_ than between *∆F*_*L&H0*_ and *∆F*_*YAN*_, *∆F*_*VR1*_ and *∆F*_*VR2.*_ When only groups of generations (initial, intermediate and most recent generations) were considered, correlations between individual *∆F* values were generally lower than when all generations were used (Supplementary Table S3). The strongest correlations were detected between *∆F*_*L&H0*_ and *∆F*_*NEJ0*_(1.00) in all generation groups. In summary, the molecular *∆F* with the strongest correlations among the others in the different generation groups was *∆F*_*YAN,*_ followed by *∆F*_*ROH0*_. Nevertheless, when only a few generations were used to calculate *∆F*, correlations between molecular and pedigree measures were very low.

**TABLE 3:** Correlation coefficients between molecular and pedigree increases in inbreeding coefficients (*∆F*) of all genotyped individuals. *∆F*_*PEDi*_ was derived from pedigree referenced against the number of generations of the individual. *∆F*_*PEDt*_ was derived from the pedigree referring to the inbreeding coefficient of the individual’s parents. *∆F*_*NEJ*_ was derived from the diagonal of the Nejati-Javaremi matrix. *∆F*_*NEJ0*_ was derived from inbreeding coefficients (*F*) obtained using the Nejati-Javaremi matrix (*F*_*NEJ*_) adjusted for the mean *F*_*NEJ*_ of the first selection generation. *∆F*_*L&H*_ was derived from the diagonal of the Li and Horvitz matrix. *∆F*_*L&H0*_ was derived from *F* using the Li and Horvitz matrix (*F*_*L&H*_) adjusted for the mean *F*_*L&H*_ of the first selection generation. *∆F*_*VR1*_ was derived from the diagonal of the Van Raden type 1 matrix. *∆F*_*VR2*_ was derived from the diagonal of the Van Raden type 2 matrix. *∆F*_*YAN*_ was derived from the diagonal of the Yang matrix. ∆*F*_*ROH*_ was computed using runs of homozygosity. *∆F*_*FROH0*_ was derived from *F* obtained using runs of homozygosity (*F*_*ROH*_) adjusted for the mean *F*_*ROH*_ of the first selection generation.

	*∆F*_*PEDi*_	*∆F*_*PEDt*_	*∆F*_*NEJ0*_	*∆F*_*L&H*_	*∆F*_*L&H0*_	*∆F*_*VR1*_	*∆F*_*VR2*_	*∆F*_*YAN*_	*∆F*_*ROH0*_
*∆F*_*PEDi*_	1.00	0.38	0.41	0.73	0.41	0.45	0.48	0.68	0.39
*∆F*_*PEDt*_		1.00	0.21	0.26	0.21	0.18	0.18	0.25	0.23
*∆F*_*NEJ0*_			1.00	0.79	1.00	0.53	0.41	0.69	0.91
*∆F*_*L&H*_				1.00	0.79	0.69	0.61	0.92	0.72
*∆F*_*L&H0*_					1.00	0.53	0.41	0.69	0.91
*∆F*_*VR1*_						1.00	0.96	0.89	0.43
*∆F*_*VR2*_							1.00	0.86	0.33
*∆F*_*YAN*_								1.00	0.61
*∆F*_*ROH0*_									1.00

Table 4 shows *Ne* calculated via different methodologies with molecular and pedigree data across the selection generations. The *Ne* trend stabilises after the 10th generation of selection except for *Ne*_*PEDt*_, which presented high variability across generations. In fact, *Ne*_*PEDt*_ showed high values in generations 11 (1,482.27) and 26 (1,138.82) and a negative value in the second generation (−80.03). The *Ne*_*PEDi*_ showed neither values as high as *Ne*_*PEDt*_ nor negative values. The *Ne*_*PEDi*_ showed the highest values in the first generations (94.72 in the first, 143.44 in the second, and 129.46 in the third). The lowest value of *Ne*_*PEDi*_ was identified in the 25th generation (34.37). The mean and standard deviation across generations for *Ne*_*PEDt*_ were 125.94 and 346.28, respectively, and 53.58 and 28.38, respectively, for *Ne*_*PEDi*_. Regarding molecular measurements, *Ne* performance differed across methodologies. Extremely negative values were detected with *Ne*_*L&H*_ in the first five generations of selection, the most negative being in the fifth generation (−4,081.60). These extremely negative values translated into a negative mean value (−228.68) with a high standard deviation (988.62). The *Ne*_*NEJ0*_, *Ne*_*L&H0*_, *Ne*_*ROH0*_, and *Ne*_*YAN*_ also presented negative values in the first and second generations. The *Ne*_*VR2*_ had lower values compared with other molecular approaches, with a mean of 23.26 compared with 35.87 for *Ne*_*NEJ0*_, 35.88 for *Ne*_*L&H0*_, 32.66 for *Ne*_*VR1*_, 40.76 for *Ne*_*YAN*_ and 30.48 for *Ne*_*ROH0*_. The standard deviation of *Ne*_*VR1*_ was the lowest (6.11). The evolution of *Ne*_*NEJ0*_ and *Ne*_*L&H0*_ was almost identical. The evolution of *Ne*_*NEJ0*_, *Ne*_*L&H0*_, *Ne*_*VR1*_, *Ne*_*YAN*_, and *Ne*_*ROH0*_ as of the 10th generation was similar to that of *Ne*_*PEDi*_, while *Ne*_*L&H0*_ showed greater values than *Ne*_*PEDi,*_ and *Ne*_*VR2*_ showed lower values than *Ne*_*PEDi*_. The evolution of *Ne*_*EXP*_ was more irregular than that of *Ne*_*NEJ0*_, with extremely negative and positive values, such as 1852.44 in the 16th generation and −464.38 in the 26th generation (Supplementary Figure S1). When selected lines were analysed separately, *Ne*_*EXPH*_ presented a more regular evolution than *Ne*_*EXPL*_. However, extremely negative and positive values were also observed in both (Supplementary Figure S1).

**TABLE 4:** Evolution across selection generations, total mean, and standard deviation (SD) of the effective population size (*Ne*) using pedigree and molecular information. *Ne*_*PEDi*_ derived from the individual increase in inbreeding (*∆F*) refers to the number of individual generations. *Ne*_*PEDt*_ derived from *∆F* based on the increase in pedigree inbreeding between two successive generations. *Ne*_*NEJ0*_ derived from the inbreeding coefficient (*F*) of the Nejati-Javaremi genomic matrix (*F*_*NEJ*_) adjusted for the mean *F*_*NEJ*_ of the first generation of selection. *Ne*_*L&H.*_ derived from *∆F* of the Li and Horvitz genomic matrix. *Ne*_*L&H0*_ derived from *F* of the Li and Horvitz genomic matrix (*F*_*L&H*_) adjusted for mean *F*_*L&H*_ of the first generation of selection. *Ne*_*VR1*_ derived from *∆F* of the Van Raden type 1 genomic matrix. *Ne*_*VR2*_ derived from *∆F* of the Van Raden type 2 genomic matrix. *Ne*_*YAN*_ derived from *∆F* of the Yang genomic matrix. *Ne*_*ROH0*_ derived from the *F* of runs of homozygosity (*F*_*ROH*_) adjusted for mean *F*_*ROH*_ of the first generation of selection.

Generation of selection	*Ne*_*PEDi*_	*Ne*_*PEDt*_	*Ne*_*NEJ0*_	*Ne*_*L&H*_	*Ne*_*L&H0*_	*Ne*_*VR1*_	*Ne*_*VR2*_	*Ne*_*YAN*_	*Ne*_*ROH0*_
1	94.72	43.63	-	−142.61	-	-	-	-	-
2	143.44	−80.03	−59.10	−118.03	−59.06	10.06	7.78	163.56	−66.05
3	129.46	99.66	−60.91	−104.36	−60.82	62.69	34.01	−69.23	−99.30
4	89.12	28.62	73.27	−3,145.47	73.29	28.65	21.61	76.17	57.87
5	66.21	20.37	96.57	−4,081.60	96.48	38.53	24.19	76.73	66.90
6	59.57	26.12	56.56	246.04	56.56	37.62	27.47	59.01	45.51
7	51.89	23.14	42.17	121.14	42.17	27.48	18.84	36.25	34.58
8	51.62	41.49	51.29	139.96	51.28	30.47	21.02	41.52	46.08
9	47.54	25.00	38.58	85.32	38.58	26.35	19.40	33.78	35.16
10	44.54	25.00	45.05	95.94	45.04	31.24	21.99	39.16	42.64
11	47.42	1,482.27	52.82	109.82	52.81	36.05	24.02	43.04	48.79
12	42.20	15.78	38.09	69.21	38.08	29.44	21.30	33.77	36.58
13	41.58	33.55	45.07	80.78	45.06	35.44	25.74	41.10	41.98
14	40.84	30.51	40.63	68.67	40.62	34.90	25.25	38.10	40.13
15	40.11	30.65	37.57	60.70	37.57	32.57	23.83	34.96	35.71
16	38.69	23.75	36.51	57.11	36.51	30.43	22.31	32.69	35.33
17	38.74	43.40	37.79	57.88	37.81	35.05	25.66	36.26	37.08
18	38.18	28.85	35.51	52.78	35.51	31.90	23.92	33.23	35.63
19	36.88	21.92	36.52	53.34	36.51	31.86	23.66	33.63	36.20
20	37.34	45.44	40.55	58.79	40.56	34.56	24.95	36.57	39.11
21	36.59	26.49	35.33	49.69	35.33	31.47	23.42	32.74	35.54
22	35.74	23.29	34.48	47.68	34.48	31.47	23.78	32.54	34.61
23	35.64	28.25	34.40	46.95	34.42	31.49	23.63	32.29	33.92
24	35.02	24.01	37.40	50.77	37.44	33.31	25.18	34.91	36.77
25	34.37	24.52	34.71	46.28	34.73	32.01	24.40	32.99	34.77
26	35.65	1,138.82	35.93	47.50	35.94	31.43	24.20	33.26	36.49
Mean	53.58	125.94	35.87	−228.68	35.88	32.66	23.26	40.76	30.48
SD	28.38	346.28	31.59	988.62	31.57	10.09	6.11	35.30	34.40

The *Ne*_*s*_ was 85.33, which was used to infer the number of generations that had passed from the founder population from the hypothetical foundation to the first generation of selection using *F*_*NEJ*_ (*t*_*NEJ*_) and *F*_*ROH*(_ *t*_*ROH*)_ as described above: *t*_*NEJ*_ was 162 generations, and *t*_*ROH*_ was 133 generations.

When pedigree data was used to calculate the number of generations elapsed if the breeding method of the selection experiment would be applied from the founder population of the recorded pedigree to the first generation of the experiment, *t*_*PED*_ was two generations.

## 4 Discussion

This population, formed by two divergent lines selected for birth weight variability during twenty-six discrete generations, allowed us to test different inbreeding measures using molecular and pedigree data and their application to compute *Ne*.

The inbreeding coefficient has been defined in terms of correlations between parents uniting gametes (Wright, 1931) and as a probability that two genes randomly sampled in the parents’ gametes are IBD (Malecot, 1948). Because of *F*_*PED*_ is completely defined in terms of IBD probability, the performance of the molecular inbreeding coefficients are going to be compared with *F*_*PED*_, although the use of *F*_*PED*_ as the golden standard is not straightforward as assumes that the founders of the pedigree are unrelated (Keller et al.*,* 2011) and provide the expected proportion of inbreeding while ignoring the effects of other forces such as Mendelian sampling, linkage, or natural selection against homozygous alleles (Wright, 1931). The *F*_*L&H*_ *, F*_*VR1*_ *, F*_*VR2*_, and *F*_*YAN*_ behave more like correlations and have negative values in some individuals (Supplementary Table S1), as reported by other authors (Alemu et al., 2021; Villanueva et al., 2021). In the ROH definition, a long stretch of homozygosity is expected to be inherited from a common ancestor (Ceballos et al., 2018); *F*_*ROH*_ values range from 0 to 1 (Alemu et al., 2021) and present the highest correlations with *F*_*PED*_ when all individuals from these populations are used. Therefore, among all the different measures of molecular inbreeding analysed in this population, *F*_*ROH*_ better fits the IBD definition when *F*_*PED*_ is used as the reference coefficient. However, ROH analysis is an empirical, rule-based approach, and detection relies on the definition of several parameters that must be adjusted as a function of several constraints, such as marker density or the number of genotyping errors (Ferenčaković et al*.,* 2013; Meyermans et al., 2020). Hence, ROH detection varied across different studies. Moreover, *F*_*ROH*_ is more representative of IBD than *F*_*NEJ*_, defined as the proportion of homozygous SNPs in the individual and, thus, unable to differentiate between IBD and identity by state (IBS) (Toro et al*.,* 2014); however, *F*_*ROH*_ theoretically represents the proportion of long IBS regions in the genome. Furthermore, if *F*_*ROH*_ and *F*_*NEJ*_ were used to infer the number of generations that had passed since the founder population, *t*_*NEJ*_ would be expected to present the highest value due to *F*_*NEJ*_ being fully IBS, while *F*_*ROH*_ is expected to be more representative of IBD. The high values of *t*_*PED*_ and *t*_*ROH*_ reflected the history of the founder population originating from the genetic contribution of three inbred mouse lines: BALB/c, C57BL and CBA. This mixed starting population was maintained under panmixia for 40 generations before the start of the selection experiment (Formoso-Rafferty et al*.,* 2016b). The lowest correlations between *F*_*PED*_ and molecular inbreeding coefficients were detected with *F*_*VR1*_ and *F*_*VR2*._ In addition, other authors (Caballero et al., 2022) reported that the poorest performance with regard to IBD was detected for *F*_*VR2*_.

The number of generations that had passed before starting the experiment inferred from pedigree (*t*_*PED*_) was very low. Pedigrees, however, are usually limited to a few past generations and ignore some of the ancestral relationships (Keller et al.*,* 2011). In this case, *F*_*PED*_ was calculated using five generations before initiating the experiment, and the mating method was changed at this start; therefore, *t*_*PED*_ was lower than the number of generations that had passed since the founder population in the pedigree.

When all data was used, correlations between different inbreeding measures were high. In other studies in Iberian pigs (Saura et al., 2015), cattle (Alemu et al., 2021; Lozada-Soto et al., 2022) or humans (McQuillan et al., 2012), strong correlations were also detected. However, when a low number of generations was used, the correlations between different pedigrees and genomic inbreeding measures were lower. Therefore, the high correlations observed when using all animals across generations were probably because of the large number of genotypes distributed across generations, resulting in a wide range of inbreeding values in all coefficients. This demonstrates that this experimental population was of particular interest to check the relationship between the different parameters assayed. Moreover, other authors reported that the incompleteness of the pedigree should lead to poor correlations (Schiavo et al., 2022). Furthermore, the correlations between the different increases in inbreeding were lower than between the inbreeding coefficients because, firstly, *∆F*_*PEDi*_ and *∆F* molecular are standardised by the depth of pedigree information. Therefore, the evolution of *∆F* was expected to be stable if the mating design remains stable across generations, generating less variation than in the case of inbreeding coefficients and lower correlations. In addition, *ΔF*_*PEDi*_ was also expected to correlate better with the other coefficients than *∆F*_*PEDt*_, because the estimating molecular ∆Fs were designed following *∆F*_*PEDi*_. We could not fit the *∆F*_*PEDt*_ formula to the molecular information because we did not genotype the trios.

If the frequencies of the base population are known, the performances of *F*_*L&H*_, *F*_*VR1*_, *F*_*VR2*_, and *F*_*YAN*_ are in terms of IBD (Caballero et al., 2022). However, other authors have reported that *F*_*L&H*_, *F*_*VR1*_, *F*_*VR2*_, and *F*_*YAN*_ presented values outside of Malecot and Wright’s inbreeding definitions and that *F*_*NEJ*_ better fitted these definitions, as its values ranged between 0 and 1 (Saura et al., 2015; Villanueva et al., 2021). Moreover, Villanueva et al. (2021) observed that only *F*_*L&H*_ could be interpreted in terms of loss or gain of genetic variability, while *F*_*VR1*_, *F*_*VR2*_, and *F*_*YAN*_ presented some inconsistencies concerning loss or gain of genetic variability. Regarding the application of inbreeding measures, *F*_*ROH*_ and *F*_*PED*_ should be preferred to measure the increase in whole genome homozygosity, and *F*_*ROH*_ should be preferred over *F*_*PED*_ when pedigrees are not deep enough or present many errors (Keller et al., 2011; Alemu et al., 2021).

It has been reported that *F*_*YAN*_ and *F*_*VR2*_, giving higher weight to rare alleles (Villanueva et al.*,* 2021), presented the highest correlation with homozygosity at SNPs with moderate to high MAFs (Alemu et al., 2021). However, alleles with low MAFs are more representative of kinship because of the higher probability of being transmitted when individuals belong to the same family. Hence, *F*_*PED*_ and *F*_*ROH.*_ did not reflect the segregation of these low-frequency alleles that could be an indicator of kinship. Therefore, to measure global inbreeding, *F*_*PED*_ and *F*_*ROH*_ were more useful (Alemu et al., 2021; Caballero et al., 2022), and *F*_*YAN*_ or *F*_*VR2*_ could be better for measuring population kinship.

The *F*_*L&H*_ represents a modification of *F*_*NEJ*_ adjusted for the expected homozygosity of the reference population. Hence, these inbreeding coefficients have negative values in some individuals (Supplementary Table S3). For *F*_*L&H*_, this is clearly represented in Figure 1, where expected homozygosity was greater than *F*_*NEJ*_ during the first ten generations. This was reflected as negative values of the individual increases in inbreeding and high negative and positive *Ne*_*L&H*_ values during the first eleven generations. The *F*_*L&H*_ is designed using the allele frequencies of a reference population to express homozygosity in terms of IBD, such as *F*_*VR1*_, *F*_*VR2*_, and *F*_*YAN*_. However, *∆F*_*L&H*_ presented more variability during the first five generations of selection (Figure 3) than *F*_*VR1*_, *F*_*VR2*_, and *F*_*YAN*_. This was probably because *F*_*L&H*_ had a higher influence on the change in the mating design during the first selection generations and was more dependent on Hardy-Weinberg disequilibrium. This problem was solved when *F*_*L&H*_ was adjusted for the mean inbreeding coefficient of the first generation of selection (*Ne*_*L&H0*_). Arias et al. (2023) described that the adjustment of the inbreeding coefficients by the mean of the reference population did not allow a complete correction of the estimates, however, the use of the allele frequencies of the RP to estimate the IBD evolution in the population studied hides some assumptions, as the reference population is in Hardy-Weinberg equilibrium or that the RP have no molecular kindship, as it is assumed to be the real founder population. Therefore, adjusting the molecular inbreeding estimates for the mean inbreeding of the RP brings these estimates closer to the IBD definition. In this context, *F*_*ROH*_ and *F*_*NEJ*_ presented the advantage of not being influenced by the allele frequencies of the RP in their estimation and of being easily corrected by the mean *F*_*ROH*_ and *F*_*NEJ*_ of the RP. Furthermore, the first two generations presented negative values for *Ne*_*NEJ0*_, *Ne*_*L&H0*_, and *Ne*_*ROH0.*_ due to a negative individual increase in inbreeding produced by a negative trend of inbreeding coefficients in the first two generations, which was probably produced by the abrupt change in mating design. Thus, this negative trend could also be because in the five previous generations before the experiment, individuals were randomly mated, and from the first generation of selection, matings were designed to avoid sharing grandparents to avoid inbreeding. In addition, none of the effective population sizes started to present stable values until the 10th generation. Toro et al. (2020) had previously pointed out that when optimal management of genetic diversity is implemented in a population, molecular estimates of *Ne* could be meaningless because the increase in genetic diversity resulted in negative *Ne*. In addition, none of the effective population sizes began to present stable values until the 10th generation. Therefore, in this population, ten generations of selection were needed to reflect the change in mating method by the effective population size, as expected, since *Ne* is a diversity indicator strongly influenced by the change in mating policy, as seen in the results of this study (Table 3).

The *Ne*_*NEJ0*_ and *Ne*_*L&H0*_ presented almost identical values in this population and similar values to *Ne*_*PEDi*_. *Ne*_*NEJ0*_ presented the advantage of not using the frequencies of a reference population such as *Ne*_*FROH0*_ and, therefore, are less dependent on allele frequencies.

Comparing *Ne*_*PEDi*_ and *Ne*_*PEDt*_, the latter presented a higher standard deviation, as other authors reported that *Ne*_*PEDt*_ presented high fluctuations over time due to the high impact of breeding method changes, errors in pedigree registration, or sampling effects (Gutiérrez et al., 2008; Gutiérrez et al., 2009; Cervantes et al., 2011; Leroy et al., 2013) and could lead to negative values (Groeneveld et al., 2009; Leroy et al., 2009; Nagy et al., 2010). The *Ne*_*PEDi*_ values were high during the 10th–12th first generations. After that, the evolution of *Ne*_*PEDi*_ tended to be stable and reflected the history of mating. Moreover, no negative values were observed, and the standard deviation was lower. Furthermore, *Ne*_*PEDi*_ evolved to be similar to the number of males of females mated in the selection experiment, accounting for the intensity of selection that could be influencing the evolution of homozygosity in this population, when the weight of the random mating previous to the experiment decreased. Another study performed in French Angora rabbits divergently selected for total fleece weight, showed that the low line had high *Ne* values calculated from pedigree data. However, this line always showed lower levels of inbreeding than the high line over the years of selection, even though the mating policy was the same in both lines (Rafat et al.*,* 2009). This did not occur in our selection lines, both of which had almost the same level of inbreeding since the beginning of the experiment (results not shown).

The minimum viable population size thresholds have been traditionally defined as 50 for the short term and 500 for the long term (Harmon and Braude, 2010). All *Ne* values of the last generations obtained for this population were lower than the defined thresholds. Low *Ne* levels relate to a decrease in genetic variability, allele fixation, and a reduction in the selection response (Domínguez-Viveros et al., 2020). Nevertheless, the use of a strict critical level for *Ne* is not straightforward, as different factors could affect its interpretation, such as the method used, the species, or population structure (Leroy et al., 2013; Mokhtari et al., 2015). Moreover, in other studies, effective population sizes below 50 were reported in some species that did not present viability problems (Leroy et al., 2013). Therefore, this threshold should be revised and adapted to the particularity of each population.

## 5 Conclusion

The results obtained in these studies allowed to understand better the performance of different inbreeding coefficients and effective population sizes applied in a population with many discrete generations and under strong selection. Moreover, these results could be used as a reference for the study of inbreeding and genetic diversity in other populations. The *F*_*ROH*_ and *F*_*PED*_ presented the strongest correlations and were more representative of inbreeding in terms of IBD. However, other measures such as *F*_*YAN*_ were useful in terms of rare alleles, which are more likely inherited from common ancestors. Adjusting the inbreeding estimates to the mean inbreeding of the reference population allowed it to fit more closely to IBD. The *Ne*_*PEDi*_ presented a more reliable performance than *Ne*_*PEDt*_. In general, *Ne* obtained by molecular data showed a similar trend when comparing molecular approaches to each other and *Ne*_*PEDi*_, being more similar to *Ne*_*L&H0*_ and *Ne*_*NEJ0*_. However, data from several generations was necessary to reach a stable trend for *Ne*, both with pedigree and molecular data.

## Data Availability

The data presented in the study are deposited in the Figshare repository, accession link: https://figshare.com/s/632fbaefccc501da66e1.
