# Supplementary figures and images for: Genomic inbreeding measures applied to a population of mice divergently selected for birth weight environmental variance
Source: Front Genet. 2023 Dec 14;14:1303748. doi: 10.3389/fgene.2023.1303748 (PMC10752941; doi:10.3389/fgene.2023.1303748)

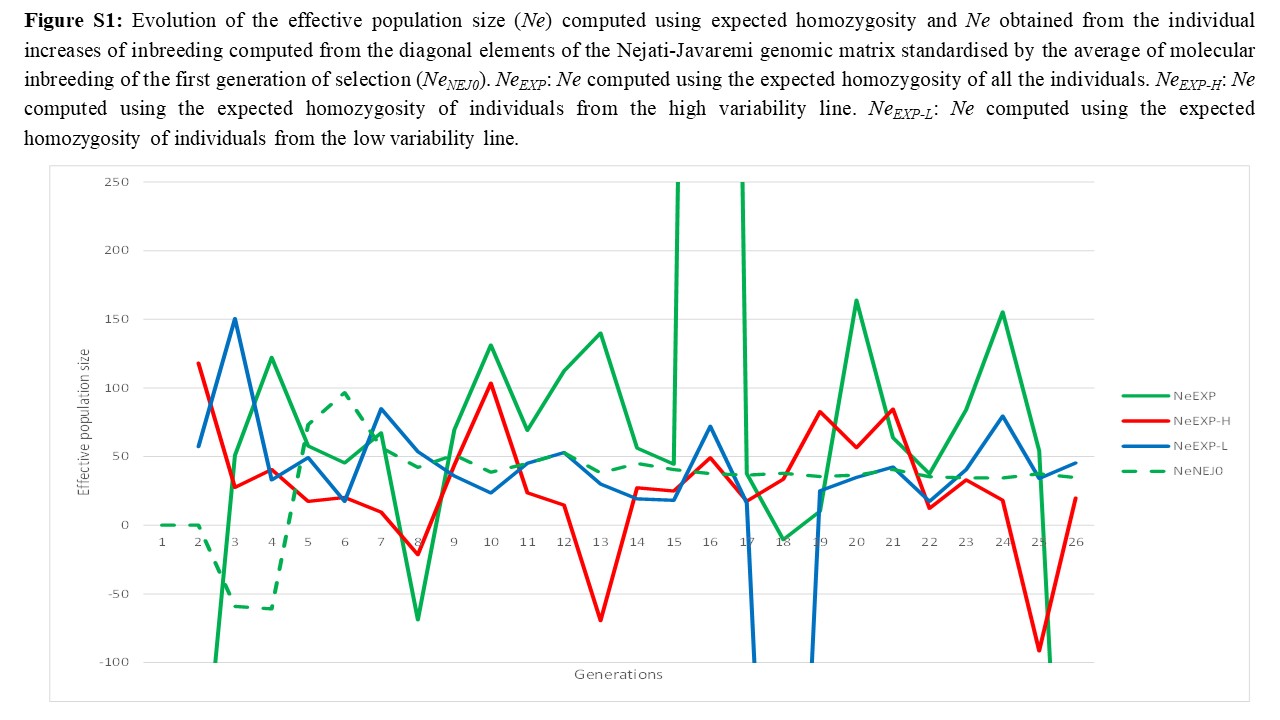

Supplement: Supplementary file 3 [file Image1.JPEG]
